# Social-structural contexts of needle and syringe sharing behaviours of HIV-positive injecting drug users in Manipur, India: a mixed methods investigation

**DOI:** 10.1186/1477-7517-8-9

**Published:** 2011-05-13

**Authors:** Venkatesan Chakrapani, Peter A Newman, Murali Shunmugam, Robert Dubrow

**Affiliations:** 1 Indian Network for People Living with HIV/AIDS (INP+), 41 (Old # 42/3), Second Main Road, Kalaimagal Nagar, Ekkaduthangal, Chennai-600097, India; 2 University of Toronto, Factor-Inwentash Faculty of Social Work, 246 Bloor Street West, Toronto, Ontario, M5S 1A1, Canada; 3 Yale University, Yale School of Public Health, 60 College Street, P.O. Box 208034, New Haven, CT, United States

## Abstract

**Background:**

Few investigations have assessed risk behaviours and social-structural contexts of risk among injecting drug users (IDUs) in Northeast India, where injecting drug use is the major route of HIV transmission. Investigations of risk environments are needed to inform development of effective risk reduction interventions.

**Methods:**

This mixed methods study of HIV-positive IDUs in Manipur included a structured survey (n = 75), two focus groups (n = 17), seven in-depth interviews, and two key informant interviews.

**Results:**

One-third of survey participants reported having shared a needle/syringe in the past 30 days; among these, all the men and about one-third of the women did so with persons of unknown HIV serostatus. A variety of social-structural contextual factors influenced individual risk behaviours: barriers to carrying sterile needles/syringes due to fear of harassment by police and "anti-drug" organizations; lack of sterile needles/syringes in drug dealers' locales; limited access to pharmacy-sold needles/syringes; inadequate coverage by needle and syringe programmes (NSPs); non-availability of sterile needles/syringes in prisons; and withdrawal symptoms superseding concern for health. Some HIV-positive IDUs who shared needles/syringes reported adopting risk reduction strategies: being the 'last receiver' of needles/syringes and not a 'giver;' sharing only with other IDUs they knew to be HIV-positive; and, when a 'giver,' asking other IDUs to wash used needles/syringes with bleach before using.

**Conclusions:**

Effective HIV prevention and care programmes for IDUs in Northeast India may hinge on several enabling contexts: supportive government policy on harm reduction programmes, including in prisons; an end to harassment by the police, army, and anti-drug groups, with education of these entities regarding harm reduction, creation of partnerships with the public health sector, and accountability to government policies that protect IDUs' human rights; adequate and sustained funding for NSPs to cover all IDU populations, including prisoners; and non-discriminatory access by IDUs to affordable needles/syringes in pharmacies.

## Background

Injecting drug users (IDUs) are among the highest priority subpopulations for HIV prevention identified by the National AIDS Control Organization (NACO) in India [1]. Sexual transmission is the primary route of HIV transmission across India. In Northeast India, however, injecting drug use is the major route of HIV transmission [2]. Manipur, a small state in Northeast India with a population of about 2.3 million, is among the Indian states with the highest HIV prevalence, with 1.39% of women attending antenatal clinics found to be HIV-infected [3]. The cumulative number of HIV-positive cases reported in Manipur from the start of the epidemic to May 2008 was 29,602 cases; of these, 42.1% were categorized as having contracted HIV through injecting drug use (personal communication with Manipur State AIDS Control Society, 2008). In 1998, the estimated number of IDUs in Manipur was 15,000-20,000 [4]; estimated HIV seroprevalence among IDUs in 2006 was 19.8% [3].

Manipur lies adjacent to the Golden Triangle, where the borders of Myanmar, Laos and Thailand meet; most of its eastern boundary is formed by Myanmar, the second largest opium producer in the world [5]. Manipur is on a major drug-trafficking route from the Golden Triangle; thus, illicit drugs are commonly available. Heroin, locally known as "number four" among IDUs, is considered to be the major injecting drug used in Manipur [6,7], although a powder form of dextropropoxyphene (from capsules) is also increasingly used by IDUs for injection [8,9].

Insurgency movements in Manipur and a "cold war" among ethnic groups (such as Meitei, Kuki, Paite and Naga) intermittently erupt in violent clashes [10,11] involving the government and 39 armed militant groups [12]. These have led to strict law enforcement by police and a strong military and paramilitary presence in Manipur [11,13,14]. In India, narcotic substances, such as opium, coca leaf and psychotropic substances specified in the Narcotic Drugs and Psychotropic Substances (NDPS) Act of 1985, are illegal. We have previously documented police interference in HIV prevention and care programmes among IDUs in Manipur [13].

Despite the epidemic among IDUs in Manipur and the government's focus on HIV prevention among this population, there has been limited investigation in Manipur of IDUs in general, or of HIV-positive IDUs, in particular. Risk behaviours among HIV-positive IDUs pose dangers to themselves, including hepatitis B virus (HBV) and hepatitis C virus (HCV) infection, re-infection with new HIV and HCV subtypes, and super-infection with antiretroviral drug-resistant HIV strains, as well as dangers of transmission of HIV, HBV and HCV to their sexual and drug-using partners. In fact, extremely high rates of HBV (100%) and HCV (92%) infection have been documented among HIV-positive IDUs in Manipur [15].

In the town of Churachandpur in Manipur, 98% of a sample of 191 IDUs were found to be infected with HCV and 75% to be HIV seropositive; only 7% were aware of their serostatus [6]. Most IDUs in this study (93%) reported having shared injecting equipment, while a rapid situation assessment study reported that 86% of IDUs (n = 308) in the city of Imphal, Manipur had ever shared syringes [16]. We know of no quantitative studies in Manipur, and few in India [17], that have reported on drug-related risk behaviours among IDUs by serostatus or by knowledge of serostatus.

While identifying individual-level risk behaviours and risk correlates is important, it is also crucial to explore social, economic and political factors that interact with and shape individual risk behaviours [18,19]. Several investigators have presented evidence that HIV preventive interventions for IDUs that focus only on individual behaviour change are likely to result in only partial reduction of HIV transmission risk [20-23]. Studies from many countries, such as Russia, the United States, United Kingdom, and Vietnam, have documented the importance of understanding various contexts of drug use related risk behaviours among IDUs [24-28]. In a recent comprehensive review of international literature on HIV risk among IDUs, factors identified as critical in the social structural production of risk included: cross-border trade and transport links; population movement and mixing; urban or neighbourhood deprivation and disadvantage; specific injecting environments such as prisons; social stigma and discrimination; policies, laws and policing; and complex emergencies such as armed conflict [29]. With the exception of our own work [13], we are not aware of any published studies among IDUs, including HIV-positive IDUs, in India that examine in depth the social-structural contexts of drug use related risk behaviours and how they may influence individual-level behaviours. Understanding social and structural influences on unsafe injecting drug use behaviours among HIV-positive IDUs is vital to support empirically based preventive interventions.

In the present study, we examined injecting drug use behaviours among HIV-positive IDUs in Imphal, Manipur, with a focus on the social-structural contexts of unsafe drug use and needle/syringe sharing and how these contextual factors shape individual's injecting risk behaviours.

## Methods

We conducted an integrated mixed methods investigation among HIV-positive IDUs in Imphal, including a cross-sectional quantitative survey, focus groups, and in-depth interviews. We also conducted key informant interviews with physicians treating people living with HIV (PLHIV). Data for the present study were collected as part of a larger study focused on sexual and reproductive health, as well as drug-related risk behaviours, of various subpopulations of PLHIV, one of which was IDUs in Manipur.

### Sampling and recruitment

IDUs were defined as persons who injected drugs in the 3 months before the study interview or focus group, in line with NACO's definition [30]. Other eligibility criteria were being HIV-positive for at least one year; 18 years of age or older; sexually active in the past 3 months; and able to understand and give informed consent.

Survey participants were a convenience sample recruited primarily from the Manipur Network of People living with HIV (MNP+); some were recruited from other non-governmental organizations (NGOs) that provide prevention and treatment services to IDUs in Imphal. We recruited HIV-positive IDUs for the in-depth interviews and focus groups from the same organizations. We used purposive sampling to ensure inclusion of IDUs from diverse subpopulations, such as married and single males and those who were co-infected with HBV and/or HCV.

### Data collection and analysis

For the cross-sectional quantitative survey, an interviewer-administered structured questionnaire was used to assess sociodemographic characteristics, alcohol and substance use, HIV testing and treatment, sexual behaviour and condom use, family planning and reproductive health, and sexually transmitted infections (STIs). This report focuses on sociodemographic characteristics (age, education, employment status, income, marital status and living arrangements) and alcohol and substance use. Participants were asked whether they had consumed alcohol in the past 3 months; those who reported any alcohol use were asked about days per week of alcohol use and drinks per day on the days they drank. Substance use measures included: ever used recreational drugs or injecting drugs, type of drugs used in the past 3 months, sharing of needles or syringes (yes/no) in the past 30 days, and exchange of sex for drugs/money (yes/no) in the past 30 days.

The survey questionnaire was drafted in English, translated into Manipuri, back-translated into English, and then finalized in Manipuri to ensure accuracy. Participants were interviewed in private rooms in the office of MNP+ or in other locations (such as participant's home) that were agreeable to both participants and interviewers and where privacy was assured. The average time to answer all questions was 40 minutes. Results were described using means (± standard deviation) and proportions, by gender.

For the qualitative component, we used topic guides for data collection. Topic guides for the in-depth interviews and focus groups explored methods of injecting drug use, contexts in which needle/syringe sharing or any other unsafe injecting drug use behaviours occurred after HIV diagnosis, risk reduction strategies adopted to prevent risk of HIV transmission to others, and barriers faced by participants in obtaining or using sterile needles/syringes. The topic guide for key informant interviews focused on barriers to and facilitators of safer sex and safer injecting drug use among PLHIV, and availability and quality of prevention and treatment services for PLHIV in government hospitals in Imphal. Individual interviews were about 45 to 60 minutes in duration and focus groups about 1.5 to 2 hours. All participants in the quantitative survey, in-depth interviews, and focus groups were given an honorarium of 250 Indian rupees. Key informants were not paid.

All individual interviews, focus groups and key informant interviews were audiotaped, transcribed verbatim in Manipuri, and then translated into English for data analysis. We explored interview and focus group data using a narrative thematic approach with techniques adapted from grounded theory [31,32]. Initial themes were identified using line-by-line coding. Themes were then listed, compared and contrasted by three independent researchers using a method of constant comparison [33]. We discussed the findings and interpretations at a community meeting with the field research team and IDU representatives as a form of member checking.

### Ethics and consent

The study protocol was reviewed and approved by the Research Ethics Board of University of Toronto and the Community Advisory Board of Indian Network for People living with HIV/AIDS. All participants provided informed consent. No names or any other personal identifying information was collected, and all personal identifiers were removed when audiotapes were transcribed.

## Results

### Characteristics of the participants

#### Survey

We surveyed a total of 75 IDUs, 50 males and 25 females. The mean age of participants was 35.6 ± 5.8 years for males and 31.0 ± 7.6 years for females. Seventy-six percent of men and 36% of women had completed high school. Thirty-four percent of men were unemployed, and 64% of women reported sex work as their main occupation. Almost three quarters (71%) of participants reported a monthly income of 3000 Indian Rupees or less. Of 29 men who were currently married and living with their wife, 38% reported that she was HIV-positive. All 8 women who were currently married and living with their husband reported that he was HIV-positive.

#### In-depth interviews

We conducted in-depth interviews with 4 male and 3 female IDUs. Men ranged in age from 30 to 39 years and women from 25 to 32 years. One man completed high school, and two men and two women completed elementary education. Three men were single and one married; two women were single and one married. Two men and one woman were unemployed. Two women engaged in sex work. Two key informants were interviewed, both of whom were physicians working with PLHIV in Manipur; one worked in a government hospital and the other for a non-governmental organization.

#### Focus groups

Two focus groups were conducted, one with men (n = 9) and one with women (n = 8). Men ranged in age from 28 to 48 years and women from 25 to 37 years. Six men completed high school and were currently married and living with their spouse; one was unemployed. Four women were illiterate; all but one engaged in sex work; and half were currently married and living with their spouse.

### Injecting drug and alcohol use

All 75 survey participants injected drugs in the past 3 months, and most (n = 62; 83% of men and women) injected drugs every day or most days of the week. All participants injected heroin; methamphetamine was the next most commonly injected drug (20% [n = 10] of men; 8% [n = 2] of women). One-third of participants (34% [n = 17] of men; 32% [n = 8] of women) reported sharing a needle or syringe at least once in the previous 30 days. All 17 men and 3 of the 8 women (38%) who reported sharing a needle or syringe reported sharing with at least one person of unknown HIV serostatus. Seventy-six percent (n = 13) of the men and all (n = 8) of the women who reported sharing a needle or syringe reported that they usually or always cleaned the needle or syringe with bleach before sharing. Overall, 64% (n = 16) of women, but none of the men, reported exchanging sex for drugs or money in the past three months. Three-fourths (n = 12) of the women who exchanged sex for drugs or money did so at least ten times. Seventy-four percent (n = 37) of men and 88% (n = 22) of women reported consuming alcohol in the past 3 months. Forty-four percent (n = 22) of men and 72% (n = 18) of women consumed alcohol at least once a week; 12% (n = 6) of men and 44% (n = 11) of women consumed alcohol daily.

### Contexts of needle/syringe sharing behaviours

A variety of contextual factors emerged in the qualitative data that helped to illuminate needle/syringe sharing practices among HIV-positive IDUs.

#### Fear of harassment by police and anti-drug groups

Although carrying needles/syringes is not illegal, the "stop-and-search" tactics of police lead IDUs to fear that carrying needles/syringes will constitute evidence of drug use, which is illegal, and subsequent detention by police under false charges. As a male participant explained:

While I was carrying a syringe in my pocket...I ran into some policemen frisking on the road. They found me with the syringe...they knew I was a drug user. They detained me and tried to take me to a police station. Luckily...I was let free. Then afterwards, whenever I go for drugs I never carry syringes...whatever is available at the drug peddler's place I use it. ...that is how I started sharing needles and syringes with other people.

A key informant confirmed that IDUs are often arrested by police if they are found with syringes:

[Police] know people who are drug users since they may have caught them earlier. So frisking them becomes a routine. If they are found with syringes then they are asked for money [by police]. If they can't pay, they arrest them on some false charges.

IDUs face what they described as harassment from self-professed "anti-drug" pressure groups, in addition to the police. This harassment deters many IDUs from carrying needles/syringes with them. As expressed by a male IDU:

I am also one of the drug users who share needles and syringes. Due to fear of organizations such as [....], I do not want to take the risk of carrying a syringe on my own. So it is better to go to the drug peddler's spot and whatever syringes are available at the spot, I use them.

A key informant described some of these anti-drug groups as '"parallel police" that stop and search suspected IDUs on the street:

Anti-drug groups or pressure groups stop and search people whom they suspect to be drug users - acting as a parallel-police. They also ask drug users - including youth and students - to confess in newspapers that they are drug users. Previously they used to even shoot them in the thighs...Thus they are almost like 'anti-drug *user' *groups.

#### Using injecting drugs in drug dealers' place of business (usually their home or sometimes an abandoned building): Risky micro-environment

*Lack of sterile syringes in the drug dealer's place of business*. Barriers to carrying clean needles/syringes, including fear of arrest, constrain IDUs to use unclean needles/syringes available in a drug dealers' business place, usually their home.

According to a male IDU:

In my locality drugs are easily available. Very near to this drug peddler's house....there was also a police station. For me, it is not only that the police will catch me but also my negligence. As a result I did it [with used needles/syringes] on the [drug dealer's] spot. Due to my withdrawal symptoms there even was a time when I injected it with someone's blood inside that syringe. And there was a time when my syringe fell inside the latrine and I took it out and injected with it. All these things are due to my negligence....my first priority is always given to drugs.

Thus, the severity of withdrawal symptoms, absence of sterile needles/syringes in the drug-peddler's house, and fear of being arrested by police led to the use of unclean needles/syringes in the drug-peddler's house.

##### Drug-dealers do not allow drugs to be taken out of their place of business

Drug peddlers almost always require IDUs to inject drugs at the location where they are sold, fearing that the police might learn of their business if IDUs were caught possessing their drugs. A male IDU explained: "I went to the spot but the peddlers were not allowing me to take drugs away; instead they insisted that it had to be done at the spot...I did it with the [used] syringe available there."

A male IDU described how he was compelled by the situation to share syringes with other users who were at the drug dealer's place:

If I [inject] at my home, I use the syringes we take from the NGO. But when we go to buy drugs we face tight security both from [police] commandos and the army. So I don't take syringes with me. And I can't come out with drugs from the house of the [drug] peddler. We [inject] with whatever syringe others [users] give us there.

#### Limited access to needles/syringes from pharmacies and needle and syringe programmes (NSPs.)

In Manipur, needles/syringes are sold in pharmacies. Although some IDUs reported that they buy needles/syringes from pharmacies, others reported that they have had a hard time convincing pharmacists to sell them needles/syringes. As a male IDU noted: "...most of the time, pharmacists are very strict; so we hardly buy syringes from the pharmacy. So we buy one syringe and we use it several times."

Participants described fear of being identified as an IDU by the pharmacist as a deterrent to buying needles/syringes from pharmacies. A male IDU said, "We are worried about buying syringes...[the pharmacist] might expose to other people that we are drug users. For this reason, we use old syringes." A female IDU explained: "We feel shy to ask for needles from a medical shop. They will ask so many irrelevant questions."

Some IDUs go to remote areas for injecting drugs, where there is no access to pharmacies from which they can buy needles/syringes. According to one male IDU, "We sometimes inject on top [of the hills]...to avoid police. To get a clean syringe there is not possible."

Some participants described up to tenfold mark-ups in the regular price of needles/syringes from pharmacists. A male IDU explained:

...if the syringe is five Rupees then the pharmacists will sell it anywhere from 20 to 50 Rupees [about one U.S. dollar, or about half a day's wage]. They will come to know you are asking for syringes for injecting drugs since you are buying it from them regularly and sometimes in bulk.

Several IDUs described not having any money left after buying drugs and thus being unable to purchase clean needles/syringes. A male IDU said, "After buying drugs, there would not be even one paisa [penny] left in our hands. In that case we have to use old syringes..." A female IDU reported, "We know we should not share syringes. A new syringe from the pharmacy shop is a costly affair. Who will give extra money for a syringe as such drugs are too costly?"

Finding money for buying drugs alone is a daunting task for IDUs, given their low income level. This is especially true for women who inject drugs as they may need to support their children and even other family members. Consequently, many women engage in sex work to obtain money to buy drugs, as shown in our quantitative survey. In this context, buying clean needles/syringes becomes an even more challenging proposition. As a female IDU explained:

One shot [of drugs] costs 50 Rupees...if there is no source of income then naturally for a woman this [sex work] is the easiest way to earn money...in such case she may not be interested in buying a clean syringe every time.

In Imphal city, Manipur, NSPs have been implemented by NGOs for several years. However, the absence of adequate and consistent funding from donors means that only a fraction of IDUs are covered. Consequently, some IDUs are not even aware of these programmes, especially when they reside or inject drugs in areas not covered by a program. A young woman who had recently shared syringes was surprised to hear about NSPs. She said, "What is it? I do not know. I never hear about it...It will be very nice if somebody could give me new syringes. Who will be happy in always cleaning and washing?"

Even those IDUs who have been receiving sterile needles/syringes from NSPs may not obtain an adequate number of clean needles/syringes; the supply may not match their demand, especially if they are high frequency users. A high frequency of injecting drug use also discourages IDUs from buying clean needles/syringes. A male IDU said, "I take [inject] almost daily; I could not also spend money for new syringes daily...Yes, I pick up some [syringes] at NGOs." High frequency of injecting drug use together with lack of money to pay for sterile needles/syringes and inadequate sterile needle/syringe availability from NGOs promote syringe/needle sharing among HIV-positive IDUs.

#### Unavailability of sterile needles/syringes within prisons

IDUs often end up in prison, sometimes due to actions of their own families. Some IDUs steal from their own home to purchase drugs. As a former prison inmate said, "Once I took some utensils from [my] home and sold them to buy drugs. Then I used to take other materials also. My family became suspicious and one day they found cotton and needles in my pocket. Then I told them the truth. They filed a case and sent me to jail to stop me from using drugs." A physician key informant related that family members often ask police to file false cases to send their drug-using sons to prisons in the hope that their sons will give up the "drug habit." Furthermore, he said that while some families are supportive and want to enrol their sons in drug dependence treatment centres, others disown them.

Lack of availability of sterile needles/syringes inside prisons, in spite of the availability of injecting drugs in prisons, means that sharing of needles/syringes among inmates is common. As a male IDU who had spent time in prison explained:

Inside the jail I met many of my friends. I somehow got money from my family and I started using it [drugs] inside the jail. Outside the jail I never shared syringes with others...inside the jail we do not have syringes; the police sold them for 100 rupees [more than 2 U.S. dollars) per syringe. So if I buy a syringe then I can get drugs free of cost [from friends who buy drugs]. In this way, we started sharing a syringe...sometimes we shared it after cleaning with bleach water...when police asked we told them that we used it for washing clothes.

Thus, this HIV-positive man and his fellow drug users in prison tried to reduce the risk of HIV transmission by procuring and using bleach to clean syringes.

### Risk reduction strategies adopted by HIV-positive IDUs

In spite of the variety of contextual factors that lead to needle/syringe sharing, some HIV-positive IDUs reported having adopted strategies to reduce the risk of HIV transmission to other IDUs - even if they share needles/syringes with others.

#### Being the 'last receiver' of needles/syringes and not a 'giver'

In general, participants felt that there was a silent 'don't ask, don't tell' policy - with no one telling or asking other drug users' HIV status. As a man said, "I always get syringes from others...I don't give...No, they [co-injectors] do not know [my HIV status]." In spite of nondisclosure of HIV status to other IDUs, this participant tried to avoid HIV transmission by being the 'last receiver.'

Some IDUs reported using disclosure of HIV status as an additional strategy along with being the 'last receiver' of the shared needles/syringes. As a female IDU said, "I always tell my [HIV] status to my [women] friends. So I prefer to be the last one to inject drugs." Similarly a male IDU said, "I did not know their status in the drug peddler's place...[but] I have always told them about my status. I let them inject first." This participant disclosed his HIV-positive status in a drug dealer's place in spite of the potential negative consequences such as discrimination and isolation from other users. In the community meeting and debriefing following data collection, IDUs suggested that most IDUs know that the majority of IDUs in Manipur are HIV-positive; thus other IDUs would not be shocked or react negatively even if some IDUs "announce it [HIV-positive status] in a public area using a loudspeaker."

#### Asking others to wash used needles/syringes with bleach

Recognizing that he could transmit HIV to co-injectors in the drug dealer's place, a man who usually did not disclose his HIV-positive status said, "I insist [other IDUs] to wash with bleach or water if I used the syringe first...I got this [HIV] because someone did not ask me to do so [wash syringes]..." Similarly, a male IDU who had spent time in prison reported using bleach along with his fellow prisoner drug users to clean syringes in order to prevent HIV transmission (see above).

#### 'Serosorting': Sharing needles/syringes only with other HIV-positive IDUs

Some HIV-positive IDUs share needles/syringes only with other known HIV-positive IDUs. As a male IDU said, "I give my used syringe to friends who are already [HIV-] positive."

Similarly a female IDU said that she had recently shared needles/syringes only with other female IDUs who are HIV-positive. The reasoning behind this 'serosorting' is the assumption that needle/syringe sharing between HIV-positive IDUs is not harmful, whereas they otherwise risk transmitting HIV to HIV-negative IDUs.

Although these risk reduction strategies used by HIV-positive IDUs may prevent or reduce HIV transmission to others, they place HIV-positive IDUs at greater risk of contracting HBV and HCV, as well as new HCV and HIV subtypes.

#### Withdrawal symptoms supersede concern for health

Many HIV-positive IDUs attributed sharing of or using unclean needles/syringes to the severity of their drug withdrawal symptoms. When their withdrawal symptoms begin, their need for the drug supersedes all other concerns, even as they may have knowledge of infection risks. A male IDU reported:

I know that one syringe costs only five rupees. Even though I can get them free of cost from DIC [drop-in centre of non-governmental organizations], during my withdrawal I go directly to the [drug dealer's] spot and [I use] whatever syringes are available - they are mostly already used ones. Everyone washes it with water - we did not wash it properly. It does not mean we did not realize what we have done, but the realization comes only after we had drugs.

A similar explanation was given by another man:

It [syringe sharing] is mainly due to our withdrawal symptoms ...at that very moment we forget about disease [HIV]. As a result, we are not afraid of taking the risk.

Thus, the severity of withdrawal symptoms may lead IDUs to prioritize immediate symptoms over long-term health.

## Discussion

A large proportion of HIV-positive IDUs in the present survey, conducted in Imphal city, Manipur, have adopted safer injecting drug use behaviours and do not share needles/syringes with others. However, about one-third of HIV-positive IDUs reported sharing needles/syringes at least once in the previous 30 days.

Overall, our qualitative findings illustrate how social, legal, economic, and policy contexts influence and shape individual-level injecting drug use risk behaviours of HIV-positive IDUs. Successful and effective HIV prevention and care programmes for IDUs in Northeast India may be contingent on several enabling contexts: supportive government policies on harm reduction, including in prisons; an end to harassment by the police, army, and anti-drug groups, with a combination of education for these entities about harm reduction, creation of partnerships with the public health sector, and accountability to government policies that protect IDUs' human rights; adequate funding for NSPs to cover all IDUs in an intervention area, including those who are HIV-positive, and IDUs in prisons; non-discriminatory access by IDUs to affordable needles/syringes in pharmacies; and family and societal acceptance of IDUs, including those who are HIV-positive, through family counselling and public sensitisation campaigns. Our focus groups and individual interviews with IDUs, and key informant interviews, indicated that many of these enabling contextual factors are not in place.

Buying and carrying sterile needles/syringes are not criminal activities in India [34]; however, laws criminalizing use and possession of even small amounts of recreational drugs and stringent measures taken by the police drive many IDUs underground. Many IDUs avoid buying and carrying sterile needles/syringes as possession of a needle/syringe is often taken as evidence of drug use. Studies from several other countries similarly document reluctance among IDUs to buy and carry needles/syringes due to the legal context and stringent policing practices [24,35-40]. The concentrated presence of the Indian army in Manipur due to insurgency and ethnic conflicts increases the chances that IDUs may be "frisked" and then detained if any evidence of drug use is found [13]. Similarly, drug dealers' fear of being caught by the police results in their not allowing IDUs to take drugs off site. Consequently, IDUs are forced to inject drugs at the dealers' locales with whatever needles/syringes are available, which results in sharing needles/syringes with others.

The easy availability of drugs in Manipur, a major drug trafficking route, and ongoing political insurgency, have led many NGOs ostensibly focused on development to adopt drug use and prevention as their primary goals, and to form alliances as anti-drug pressure groups. Furthermore, drug trafficking is allegedly a source of funding for some of the insurgency groups; thus combating the drug trade also serves political and military goals [41,42]. From the perspectives of participants and key informants, the actions of many NGOs and anti-drug groups often serve to produce risk by fomenting criminalization and rigid abstinence-only approaches thereby targeting drug users themselves.

Although there are free NSPs in Manipur, lack of adequate and consistent funding hinders these programmes from providing coverage to all IDUs who need them. In spite of the Indian government's recent changes in policy that now allow NSPs, delays in scaling up these programmes and failure to ensure uninterrupted funding to the NGOs that run them ultimately result in unsafe injecting drug use practices among IDUs. Furthermore, the same fear of detainment or arrest that prevents IDUs from carrying clean needles/syringes may deter utilization of NSPs. IDUs report being detained by police as they leave NSPs carrying syringes [13].

Pharmacies are another venue through which clean needles/syringes may be accessed; however, fear of being identified as an IDU prevents many IDUs from buying sterile needles/syringes in pharmacies. Additionally, pharmacists sometimes discriminate against IDUs by inflating the price of syringes, making them unaffordable.

There appears to be a confluence of factors--poverty, high costs of recreational drugs, criminal laws, harassment from anti-drug groups and police, and restrictive governmental policy on NSPs in prisons--that renders many IDUs particularly vulnerable to HIV and co-infections. Most IDUs are unemployed or have low-wage jobs. In the absence of support from family members, some IDUs engage in criminal activities in order to buy drugs, including stealing from their own families, which in turn may land them in prison-sometimes through the direct intervention of family members [13]. Although injecting drugs may be available in prisons, at even higher prices, it is very difficult to access sterile needles/syringes in prisons [13]. Consequently, many IDUs in prison, including those who are known to be HIV-positive, may be forced to share needles/syringes with others. Drug use in Indian prisons has been acknowledged by the Indian government [43]. However, India does not have government-sponsored NSPs or opioid substitution treatment programmes within prisons, which increases the spread of HIV and negatively affects the health of HIV-positive IDUs in prisons.

The most frequently cited individual-level reason for unsafe injecting drug use behaviours was the severity of drug withdrawal symptoms, which led many IDUs to prioritize immediate symptoms over long-term health. Thus, it is crucial to assist HIV-positive IDUs to treat their chemical dependency by linking them to drug substitution therapy (sublingual buprenorphine or methadone solution) and/or to drug dependence treatment programmes. NACO is presently scaling up sublingual buprenorphine substitution therapy in the third phase of the National AIDS Control Program (NACP-3) (2007-2012). Although methadone is currently illegal in India, NACO also has plans to 'pilot' methadone maintenance clinics soon [44]. This scale up should be implemented rapidly. Furthermore, methadone substitution therapy, which is not currently available in public hospitals in India, should be made available.

On the individual level, the emphasis of risk-reduction counselling for HIV-positive IDUs needs to be on avoiding needle/syringe sharing and always using clean needles/syringes. Harm reduction messages should stress not only the need to avoid risk of HIV transmission to others, but also the risk of contracting new HIV infections, including different HIV subtypes and drug-resistant strains, as well as HBV and HCV infection. However, given powerful contexts that constrain IDUs' enactment of HIV risk reduction behaviours, this counselling should be seen as only one of the steps towards providing holistic and comprehensive care to HIV-positive IDUs.

A limitation of this study was the use of a convenience sample of HIV-positive IDUs in the survey. Participants, who were recruited through MNP_+ _and other NGOs providing services to IDUs, may engage in safer injecting practices than HIV-positive IDUs who are not connected to services; risk behaviours among other HIV-positive IDUs may be even greater. Furthermore, social desirability bias may have led some participants to underreport their needle/syringe-sharing behaviours. Thus, our finding that one-third of HIV-positive IDUs shared needles/syringes may represent an underestimate. The small number of in-depth interviews and key informant interviews also represents a limitation in that we cannot ensure saturation; other participants might introduce perspectives and opinions not addressed by those who we interviewed. However, we triangulated methods (survey, in-depth interviews and focus groups) and data sources (IDU participants and key informants) to increase the validity of the findings [31].

Finally, although we identified the practices of "serosorting" in needle/syringe sharing and being the 'last receiver' of needles/syringes among HIV-positive IDUs in the qualitative component of this study, we did not measure the prevalence of these practices in the survey. Future research among IDUs should assess the prevalence of these practices among known HIV-positive and known HIV-negative IDUs. IDUs also should receive education to the effect that serosorting is not a foolproof strategy: knowledge of one's drug-using partners' serostatus may be flawed, and HCV and HBV may be transmitted regardless of HIV status.

## Conclusions

We identified a variety of powerful social, legal, economic, and policy-level factors that create a context in which HIV-positive IDUs in Manipur who might otherwise adopt safer injecting practices instead engage in needle/syringe sharing. Nevertheless, many of these contextual factors are modifiable. In addition to enhancing interventions that focus on risk reduction at the individual level, it is crucial to undertake broader structural interventions to address key contextual factors in India that, albeit unintentionally, contribute to HIV risk among IDUs. Ultimately, these higher-level interventions hold the promise of effecting sustainable reduction of HIV infections among IDUs and for improving the health of IDUs living with HIV in India.

## Competing interests

The authors declare that they have no competing interests.

## Authors' contributions

VC supervised data collection and analysis, drafted the manuscript and participated in revision of the manuscript. PAN participated in data analysis, drafting of the manuscript and led revision of the manuscript. MS carried out data collection and data analysis. RD participated in data analysis, drafting and revision of the manuscript. All authors participated in the design of the study, and read and approved the final manuscript.
